# Comments on Saint’s triad

**DOI:** 10.1186/s40792-015-0116-3

**Published:** 2015-11-14

**Authors:** Vitorino Modesto dos Santos, Lister Arruda Modesto dos Santos

**Affiliations:** Armed Forces Hospital and Catholic University of Brasília, Estrada do Contorno do Bosque s/n, Cruzeiro Novo, 70658-900 Brasília, DF Brazil; State Workers Hospital of São Paulo, São Paulo, SP Brazil

**Keywords:** Saint’s triad, Cholelithiasis, Diverticular disease, Hiatal hernia

## Abstract

Yamanaka et al. described two case studies involving coexistent cholelithiasis, hiatal hernia, and umbilical hernias, and discussed clinical similarities with the classical features of the Saint’s triad. Cholelithiasis, hiatal hernia, and colonic diverticulosis characterize the classical triad, but some authors have included any type of hernia due to herniosis—a developmental disorder of the extracellular matrix. The main features of this triad, which seem to be underdiagnosed and/or underreported, are discussed. Therefore, the commented manuscript contributed to better understanding the scarcely reported condition.

## Correspondence

Classically, the Saint’s triad includes cholelithiasis, hiatal hernia, and diverticulosis of colon [1–3]. We read the recent manuscript by Yamanaka et al. involving two case studies of overweight women who presented with clinical characteristics of the uncommon Saint’s triad, except for colonic diverticulosis [1]. Interestingly, both patients had umbilical hernias and a craniopharyngioma was diagnosed in one of them. With solid base in literature, the authors of this interesting manuscript commented historical data and classical features of the triad, and discussed the major hypotheses about its physiopathological mechanisms [1]. They also highlighted the previously described relationship of Saint’s triad with any type of hernia [2], phenomenon that probably would justify the not yet consensual use of novel terms like herniosis and Saint's tetralogy [1–3].

We consider the case studies herein commented very useful to enhancing the available knowledge about this not yet clarified and scarcely described condition, probably underrecognized or underreported.

Worthy of note, other conditions involving gastrointestinal structures, as any primary hernia [1, 2] and non-lithiasic gallbladder disorders may be associated with the occurrence of classical Saint’s triad [3, 4]. Patients with diverticulosis and gallbladder disease are more prone to have hiatus hernia (OR = 3.8, *P* = 0.0012) or any hernia (OR = 10.7, *P* < 0.0001); and chronic obstructive pulmonary disease, arterial hypertension, aortic aneurysm, and diabetes are associated with Saint's triad (including any hernia) [2].

The craniopharyngioma in the 41-year-old woman reported by Yamanaka et al., did not rule out eventual inverse correlation between herniosis and the development of other malignant tumors [3]. Changes in extracellular matrices with an increase in collagen I and fibroblasts have been detected in herniosis, allegedly considered “hostile to the development of malignancy throughout the body” [3]. Therefore, further research utilizing molecular biology tools might be done, with the purpose of clarifying this hypothetical phenomenon [3].

Although with inherent limitations of case reports, the commented article can enhance the suspicion index of primary care workers about the triad, and contributes to the knowledge of its physiopathology.

## References

[1] Yamanaka T, Miyazaki T, Kumakura Y, Honjo H, Hara K, Yokobori T (2015). Umbilical hernia with cholelithiasis and hiatal hernia: a clinical entity similar to Saint’s triad. Surg Case Rep..

[2] Hauer-Jensen M, Bursac Z, Read RC (2009). Is herniosis the single etiology of Saint’s triad?. Hernia..

[3] Read RC (2011). The Nyhus-Wantz lectureship: etiology, herniosis, diverticulosis coli, and cancer. Hernia..

[4] McAleese P, Kolachalam R, Zoghlin G (1996). Saint’s triad presenting as volvulus of the gallbladder. J Laparoendosc Surg..

